# Expression of CAMK1 and its association with clinicopathologic characteristics in pancreatic cancer

**DOI:** 10.1111/jcmm.16188

**Published:** 2020-12-20

**Authors:** Yangyang Lei, Tianzhu Yu, Changyu Li, Jianke Li, Yicheng Liang, Xinyuan Wang, Yi Chen, Xiaolin Wang

**Affiliations:** ^1^ Shanghai Institute of Medical Imaging Shanghai China; ^2^ Department of Interventional Radiology Zhongshan Hospital Fudan University Shanghai China; ^3^ Department of Thoracic Surgery National Cancer Center/National Clinical Research Center for Cancer/Cancer Hospital Chinese Academy of Medical Sciences and Peking Union Medical College Beijing China; ^4^ Institute of Immunology Department of Biology National University of Ireland Maynooth Maynooth Ireland

**Keywords:** CAMK1, pancreatic cancer, prognosis, protein‐protein interactions, tissue microarray

## Abstract

Calcium/calmodulin‐dependent protein kinase (CAMKs) can control a wide range of cancer‐related functions in multiple tumour types. Herein, we explore the expressions and clinical significances of calcium/calmodulin‐dependent protein kinase 1 (CAMK1) in pancreatic cancer (PC). The expression of CAMK1 in PC was analysed by Gene Expression Profiling Interactive Analysis 2 (GEPIA 2) database and the Oncomine database. For further validation, the protein level of CAMK1 in PC tissues was also detected in the Human Protein Atlas (HPA) database and the tissue microarray (TMA)‐based immunohistochemistry (IHC). GEPIA 2 and Kaplan‐Meier Plotter (KM Plotter) databases were used to explore the prognostic significances of CAMK1 in overall survival (OS) and disease‐free survival (DFS) of PC at mRNA level. The relationship between CAMK1 expression and the clinicopathological characteristics of PC was further explored. Additionally, the Search Tool for the Retrieval of Interacting Genes (STRING) database was used to analyse protein‐protein interactions (PPI). We found CAMK1 was highly expressed in PC both in bioinformatics analyses and TMA‐IHC results. The prognostic analyses from the public databases also showed consistent results with follow‐up data. The PPI network suggested that CALM1, CALM3, CREB1, CALM2, SYN1, NOS3, ATF1, GAPDH, PPM1F and FBXL12 were important significant genes associated with CAMK1. Our finding revealed CAMK1 has prognostic value in PC patients, suggesting that CAMK1 may has a distinct role in PC patients and can be used as a candidate marker for investigating clinical prognosis of PC.

## 1 INTRODUCTION

PC is often diagnosed at an advanced stage leaving no effective therapies. At present, the 5‐year relative survival rate of PC is about 8%, ranking lowest amongst all cancers.^1^, ^2^ The reasons for the poor survival and high mortality of PC are multi‐factorial including the close proximity of surrounding important tissues and its special tumour microenvironment.^3^, ^4^ Although surgical resection remains the only chance for cure, less than 20% of patients are even surgical candidates.^5^ In addition, despite the completion of surgical resection and adjuvant chemotherapy, nearly 60% of patients relapse within 2 years after surgery.^6^

CAMKs are serine/threonine kinases that are activated by increased intracellular calcium concentration and can mediate subsequent cell activity. Ca2 + binding greatly changes the conformation of CaM and increases its affinity for some CaMKs including CaMKK, CaMKI, CaMKII and CaMKIV. These CaM kinases are widely expressed and can participate in a variety of cancer‐related functions.^7^ Their potential in anti‐cancer treatment interventions has gradually begun to receive attention. It was reported that targeting Ca2 + signalling may provide therapeutically useful options, such as inducing epigenetic reactivation of tumour suppressor genes in cancer patients.^8^ CaMKI family consists of 4 members including CaMKIα, CaMKIβ/Pnck, CaMKIγ/CLICK3 or CaMKIδ/CKLiK, which are coded for by CAMK1, PNCK, CAMK1G and CAMK1D, respectively.^7^ CAMK1 is known to play important roles in Ca2 + signalling pathways and it is also involved in multiple cell functions, including ATP binding, signal transduction, cell differentiation, et al^9^ Despite the importance of CAMK1 in cell functions, it is also faced with some intriguing questions and challenges in tumour field. In this present study, we plan to illustrate the presence and importance of CAMK1 in PC through bioinformatic mining analysis and the samples presented in TMAs.

## 2 MATERIALS AND METHODS

### 2.1 Bioinformatics mining methods

The GEPIA 2 database (http://gepia2.cancer‐pku.cn) could analyse the gene expression profiles from the Cancer Genome Atlas (TCGA) dataset and the Genotype‐Tissue Expression (GTEx) projects. The expression level of one gene in different types of cancer could be achieved by Boxplot.^10^ We identified the expression levels of CAMK1 in PC based on TCGA normal and GTEx data. The cut‐off value of log2FC was set as 1, and *P* value was set to 0.01. Next, Oncomine (www.oncomine.org), a cancer microarray database and integrated data‐mining platform,^11^, ^12^ was used to compare CAMK1 expression in PC tissues with that in normal tissues. In this study, we chose mRNA levels of cancer vs. normal patient datasets, 1.5‐fold change and *P* value = 0.01 as threshold. We also retrieved the data from the HPA database (http://www.proteinatlas.org). The HPA database was made available freely to provide the expression profiles at protein levels, as well as IHC images for a wide variety of cancer tissues. In the HPA database, genome‐wide transcriptomics data and clinical metadata of almost 8000 patients were used in order to analyse the proteome of 17 major cancer types. The IHC analysis in the HPA database is also presented for many protein‐coding genes in respective cancer patients, the antibody information used for each IHC analysis can also be obtained in the HPA database. The IHC score is mainly classified into strong, moderate, weak and negative based on the staining intensity and fraction of stained cells.^13^, ^14^ Furthermore, we also used KM Plotter database (http://kmplot.com/analysis), an online database is capable to assess the effect of any gene on survival in cancer patients^15^ and GEPIA 2 database to evaluate the OS and DFS of PC patients. In order to assess the prognostic values of CAMK1, the patient samples were divided into two cohorts based on the median expression (high expression and low expression) of CAMK1. CAMK1 was uploaded respectively to obtain the survival plots, in which Logrank *P* value and hazard ratio (HR) with 95% confidence intervals(CI) were calculated and showed on the webpage.

### 2.2 Tissue microarray construction

TMA is a high throughput tool that allows hundreds of tissue samples to be analysed quickly, and conveniently, this method allows all tissue samples in an experiment to be analysed under standardized conditions. In our study, each TMA was constructed in the way described many times before.^16^ The sections were placed on slides coated with 3‐aminopropyltriethoxysilane. The non‐cancer tissue samples were taken at a distance of > 3 cm from the tumour margin. For TMAs detection, 90 cases of PC and matched non‐tumour tissues were obtained between January 2001 and December 2006 from Shanghai Outdo Biotech Co, LTD (TMA number: HPanA150Su01). All these human tissue samples were obtained with appropriate bioethics approvals and informed consents. Diagnoses of PC were confirmed on the basis of pathological evidence. These PC patients had not received any preoperative anti‐cancer therapy before surgery. All clinicopathological features of these PC patients were provided (Table 2), and tumour differentiation grades and clinical stages were classified based on the 7th American Joint Committee on Cancer (AJCC) TNM classification. A pathologist participated in reviewing the process.

### 2.3 Immunohistochemistry

Immunohistochemistry technology can detect antigens in tissue sections through immunological and chemical reactions, and this technique has high sensitivity and specificity and can detect a variety of antigens in tissue.^17^ We placed the paraffin‐coated microarray sections on a 60℃ heating block for 30 min and continuously washed with xylene. The slides were rehydrated in different concentrations of alcohols and boiled in a pressure cooker containing 6.5 mm sodium citrate buffer to restore the antigen.^18^ Then, we used 3% hydrogen peroxide to block the endogenous peroxidase activity for about 30 min at room temperature. Pre‐incubate the slides with bovine serum albumin (BSA) in 0.1‐mM Tris‐buffered saline (TBS) for 2 hour to reduce non‐specific background. Then we used rabbit monoclonal CAMKI antibody (ab68234, abcam) diluted 1:1000 in BSA to incubate slides at 4℃ overnight. After incubation with antibodies and BSA, we rinsed the slides with 0.05% Tween‐20 three times, 5 min each time and secondary antibody was used to incubate with the slides for 2 h at room temperature. The slides were developed in diaminobenzidine solution and stained with haematoxylin. 3 representative fields of each case were collected by Leica Aperio Image Scope software to ensure homogeneity and representativeness. The immunoreactivity score (IRS) assessments of CAMKI were performed by two independent pathologists without knowing the clinical pathological data. The immunohistochemical staining results were considered both the intensity of staining and the score for positive area. The scoring criteria for staining intensity were as follows: 0(negative), 1(weak), 2 (moderate) and 3 (strong). The criteria for the score for positive area were 0 (<10%), 1 (11‐25%), 2 (26‐50%), 3 (51‐75%) and 4 (76‐100%). Then the final expression score was calculated as the staining intensity score × positive area score, ranging from 0 to 12. A total score of 6 or higher were grouped as high expression group, and less than 6 was grouped as low expression group. The above criteria for the score were performed according to a previously described published literature.^19^

### 2.4 PPI network construction and KEGG pathway analysis

STRING database (https://string‐db.org/cgi/input.pl) can collect and integrate known and predicted protein‐protein association data. The associations in STRING database include direct (physical) interactions and indirect (functional) interactions, as long as both are specific and biologically meaningful.^20^ The identification and characterization of protein‐protein interactions will be necessary to better understand the functions and efficacy of CAMK1. In this study, we used STRING database to construct PPI network of CAMK1 with minimum required interaction score 0.7 and the interaction predictions were mainly derived from textmining, experiments, databases, co‐expression and co‐occurrence, et al The KEGG pathway analysis was also constructed by STRING database.

### 2.5 Statistical analysis

The CAMK1 expression levels between PC tissue and normal tissue were evaluated by the GEPIA 2 and the Oncomine database. The expression score in the HPA database describes a knowledge‐based best estimate of the true protein expression. The survival analyses were estimated by GEPIA 2 and KM Plotter database. The overall survival was estimated using the Kaplan‐Meier method with a logrank test. Furthermore, the variables with statistical significance in univariate analysis were included in multivariate analysis to identify independent prognostic factors by Cox proportional hazard regression model. The correlation between CAMK1 expression and clinicopathological characteristics was estimated by Chi‐square test. The *P* value <0.05 was considered as a statistical significance.

## 3 RESULTS

### 3.1 CAMK1 was highly expressed in pancreatic cancer in bioinformatics database

**FIGURE 1:**
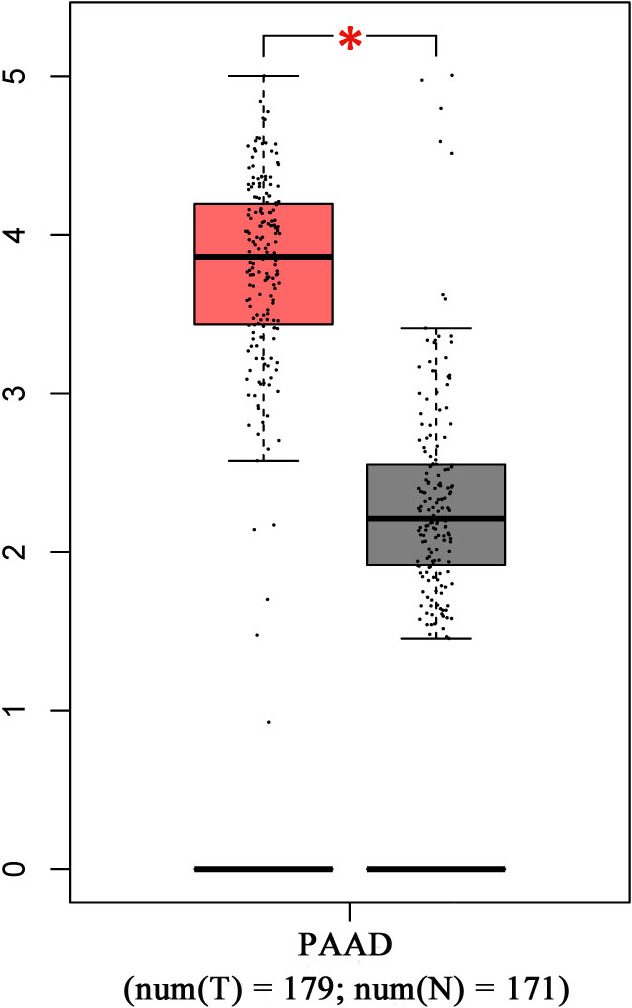
The expression of CAMK1 analysed by GEPIA 2. CAMK1 had significant expression level in pancreatic cancer specimen compared to normal specimen.*P < .01. Red colour means pancreatic cancer tissues and grey colour means normal tissues

The GEPIA 2 database was used to determine CAMK1 expression in PC and normal tissues. This results showed that CAMK1 expression was higher in PC tissue (red box) than normal tissue (grey box )(*P < .01, Figure 1). Next, the expression of CAMK1 was further validated with Oncomine database. The findings in the Oncomine database showed that CAMK1 mRNA expression was elevated in PC tissues when compared to normal tissues in the Logsdon Pancreas's dataset with the reporter L41816 (Figure S1A), the Ishikawa Pancreas's dataset with the reporter 204 392 (Figure S1B) and the Iacobuzio‐Donahue Pancreas 2’s dataset with the reporter 52 629(1) (Figure S1C). We also yielded and analysed IHC data for PC tissues from the HPA database, and the CAMK1 staining showed moderate to strong cytoplasmic immunoreactivity in most PC tissues (Antibody HPA051409) (Table 1).

**TABLE 1:** The IHC data were yielded and analysed for pancreatic cancer tissues from the Human Protein Atlas database. The CAMK1 staining showed moderate to strong cytoplasmic immunoreactivity in most PC tissues (Antibody HPA051409)

Group	The number of samples	Percentage (%)
CAMK1 staining		
High	1	9 (1 of 11)
Medium	6	55 (6 of 11)
Low	2	18 (2 of 11)
Not detected	2	18 (2 of 11)
CAMK1 intensity		
Strong	1	9 (1 of 11)
Moderate	7	64 (7 of 11)
Weak	2	18 (2 of 11)
Negative	1	9 (1 of 11)
CAMK1 quantity		
>75%	5	45 (5 of 11)
75% −25%	3	27 (3 of 11)
<25%	2	18 (2 of 11)
None	1	9 (1 of 11)
CAMK1 location		
Nuclear	0	0
Cytoplasmic/membranous	10	91 (10 of 11)
Cytoplasmic/membranous, nuclear	0	0
None	1	9 (1 of 11)

### 3.2 Predicting the prognostic values of CAMK1 in pancreatic cancer based on GEPIA 2 and KM Plotter database

**FIGURE 2:**
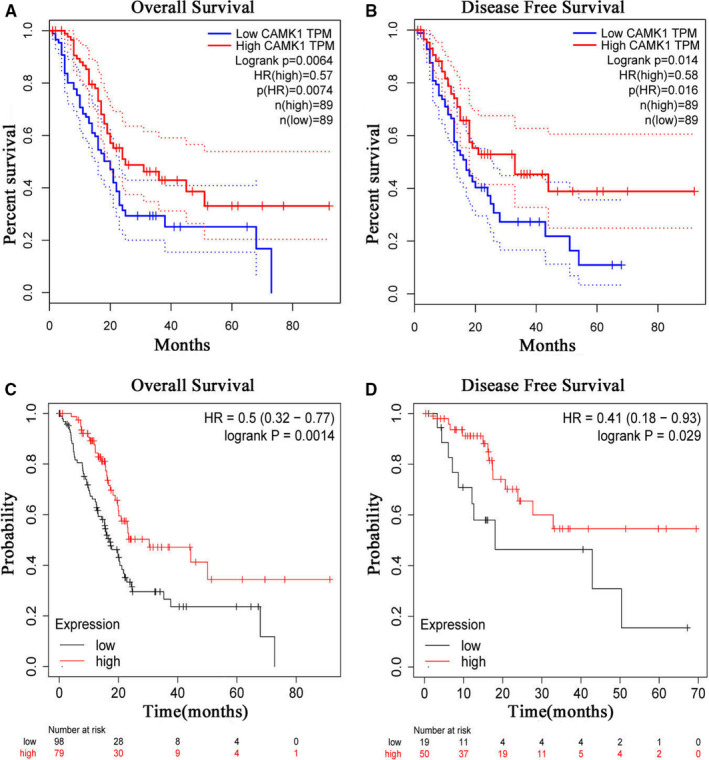
Kaplan‐Meier survival curves comparing the high and low expression of CAMK1 in pancreatic cancer (A‐D). (A‐B) Survival curves of OS and DFS in pancreatic cancer in GEPIA2 database(n=178, n=178,), high expression of CAMK1 was correlated with better OS (HR=0.57, *P*=.0074) and DFS(HR=0.58, *P*=.016) in pancreatic cancer. (C‐D) Survival curves of OS and DFS in pancreatic cancer in KM plotter database (n=177, n=69), high expression of CAMK1 was correlated with better OS (HR=0.5, *P*=.0014) and DFS (HR=0.41, *P*=.029) in pancreatic cancer, too

To better understand the relevance of CAMK1 expression in PC patients, we investigated the relationship between CAMK1 expression and clinical characteristics of PC patients in GEPIA 2 and KM Plotter database. It was found that high expression of CAMK1 was associated with better OS and DFS for PC patients in GEPIA 2 database(HR = 0.57, Logrank *P*= .0064; HR = 0.58, Logrank *P*= .014 ) (Figure 2A,B). To further investigated the prognostic potential of CAMK1 in PC, KM Plotter database was used to evaluate the CAMK1 prognostic value. Again, we found that high CAMK1 expression was correlated with better OS and DFS [HR = 0.5(0.32‐0.77), Logrank *P*= .0014; HR = 0.41(0.18‐0.93), Logrank *P*= .029] (Figure 2C,D).

### 3.3 Independent validation of prognostic value of CAMKI by TMA‐based IHC

**FIGURE 3:**
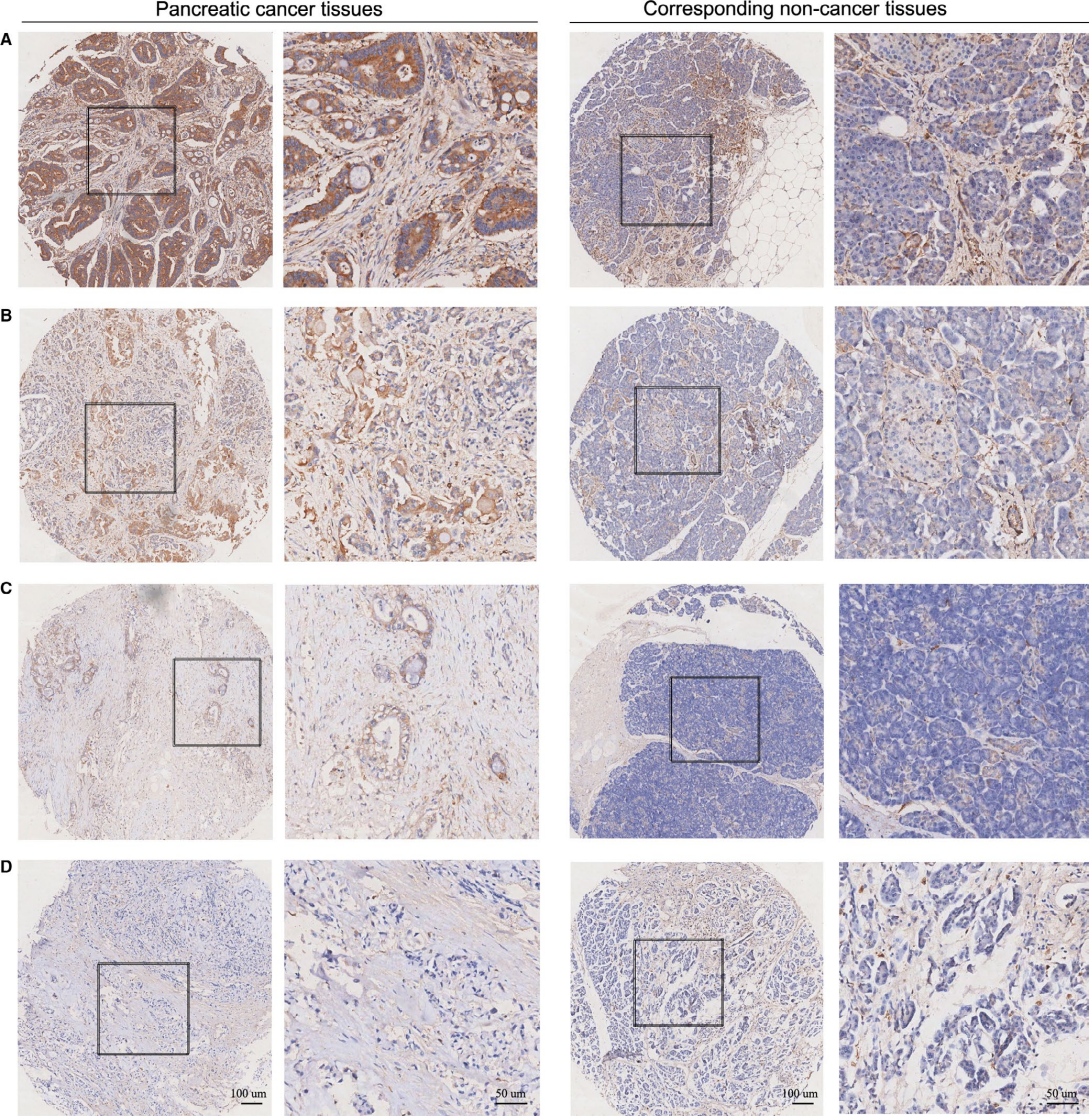
CAMK1 protein expression in PC tissue samples and corresponding non‐cancer tissue samples. CAMK1 protein levels were up‐regulated in PC tissues compared to the corresponding non‐cancer tissues in our TMA‐IHC results. CAMK1 protein showed general cytoplasmic expression in cancer cells. Representative TMA‐IHC images of different staining intensities of CAMKI (A‐D). A: strong intensity of CAMK1 in pancreatic cancer tissues; B: moderate intensity of CAMK1 in pancreatic cancer tissues; C: weak intensity of CAMK1 in pancreatic cancer tissues; D: negative intensity of CAMK1 in pancreatic cancer tissues

**FIGURE 4:**
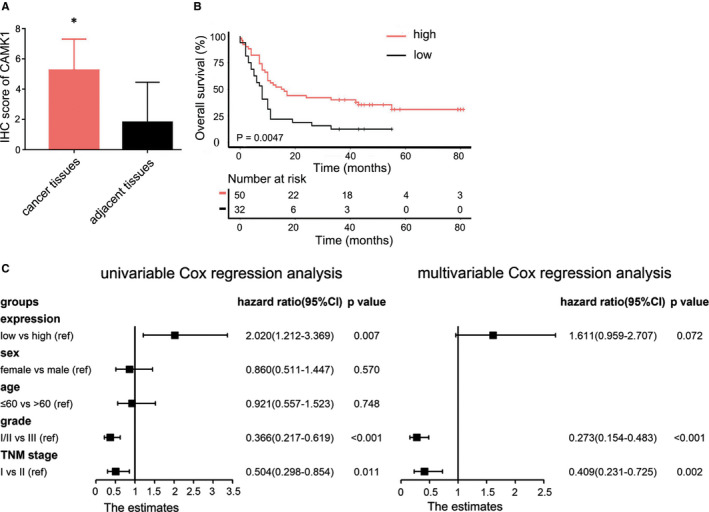
The expression of CAMK1 and their influences on OS of PC patients. (A) The expression level of CAMKI in pancreatic cancer was higher than those in corresponding non‐tumour tissues. *P*<.05. (B) Patients with CAMK1 higher expression showed a superior OS compared to patients with CAMK1 lower expression. The median OS was 15 months (95% CI 8.07‐21.93 months) in CAMK1 higher expression patients compared to a median OS of 8 months(95% CI 5.82‐10.17 months, *P*=.0047) in CAMK1 lower expression patients. (C) Cox proportional hazards model analysis of prognostic factors

Considering the results of prognostic value of CAMK1 in database, we further validated the prognostic value of CAMK1 expression by using TMA‐based IHC in 90 paired PC tissues and corresponding adjacent non‐tumour tissues. Eventually, eliminating 8 ineffective tissues, 82 PC tissues included, with 50 male and 32 female. The median age of the patients was 60 years, ranging from 83 years to 34 years. In TMA‐based IHC, CAMKI was mainly located in the cytoplasm of PC cells, and the different staining intensities of CAMKI were displayed in Figure 3. CAMKI protein levels were up‐regulated in PC tissues compared to the adjacent tissues(Figure 4A). Higher expression of CAMK1 was associated with a better OS of PC patients(median OS 15 vs. 8 months, *P*=.0047, Figure 4B). The univariate analyses indicated that CAMK1 expression, grade and TNM stage played important roles in the prognosis of PC (*P*=.007, *P*<.001 and *P*=.011, respectively). These variables with statistical significance in the univariate analyses were included in a multivariate regression analysis, and the results showed that grade and TNM stage were the significant independent prognostic factors of PC (*P*<.001 and *P*=.002, respectively), but not CAMK1 (Figure 4C). Furthermore, the association between clinicopathological variables and CAMK1 immunostaining was also analysed using Pearson's chi‐square test (Table 2). The results showed that CAMKI expression in PC may be associated with TNM stage and N stage(*P*=.013 and 0.038, respectively).

**TABLE 2:** Correlation between CAMK1 expression and clinicopathological characteristics of pancreatic cancer patients in the TMA‐IHC cohort. *P*< .05 was considered statistically significant

	variables	CAMK1		Total	χ^2^	p value
		Low	HIGH			
Age (year)					1.589	0.208
	≤ 60	18	21	39		
	>60	14	29	43		
Sex					2.62	0.106
	Female	9	23	32		
	Male	23	27	50		
Grade					0.497	0.481
	I/II	20	35	55		
	III	12	15	27		
T stage					1.355	0.244
	T1/T2	25	45	70		
	T3	7	5	12		
Tumour sizes (cm)					0.178	0.674
	≤5	19	32	51		
	>5	13	18	31		
N stage					4.305	0.038
	N0	13	32	45		
	N1	19	18	37		
TNM stage					6.123	0.013
	Ι	9	28	37		
	II	23	22	45		
Invasion of nerve, lymph or blood vessels					0.262	0.608
	No	20	34	54		
	Yes	12	16	28		

### 3.4 PPI network and KEGG pathway analysis

**FIGURE 5:**
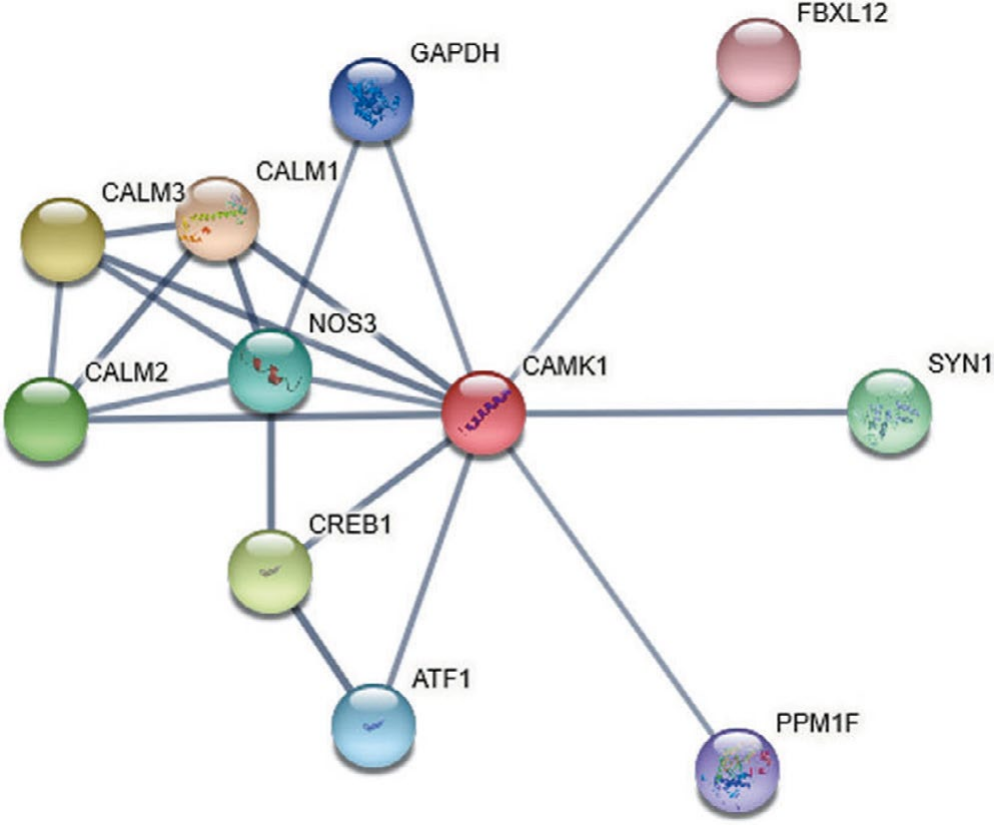
The PPI information about CAMK1 evaluated by STRING database. The predicted functional genes with CAMK1 included CALM1, CALM3, CREB1, CALM2, SYN1, NOS3, ATF1, GAPDH, PPM1F and FBXL12

The PPI information about CAMK1 can be evaluated by STRING database. A PPI network consisted of 11 nodes and 28 edges. Each node represented all the proteins produced by a single, protein‐coding gene locus and each edge represented the predicted functional associations. The predicted functional genes with CAMK1 mainly included CALM1, CALM3, CREB1, CALM2, SYN1, NOS3, ATF1, GAPDH, PPM1F and FBXL12 (Figure 5). The PPI information and pathway data enrichment analysis indicated that CAMK1 was mainly enriched in several KEGG pathways associated with aldosterone synthesis and secretion, oxytocin signalling pathway, et al (Supplementary Table 1). The candidate genes in these pathways included CALM1, CREB1, ATF1 and NOS3, and they were all up‐regulated in PC (*P*<.05, Figure S2).

## 4 DISCUSSION

Calcium is a widespread second messenger which controls various mechanisms required for cell motility. In human body, CaM transmits information to many interaction partners by sensing local changes in Ca2 + concentration. Ca2+/CaM complex can modulate the activities of enzymes, channels, signals, adaptor and structural proteins, thereby regulating the functions of related signalling pathways that control various cell functions.^21^, ^22^, ^23^ It is reported that CaM can regulate cell growth and its function may change in malignant tumours.^24^, ^25^ Changes in CaM‐dependent cell cycle and proliferation have been observed in many tumour cells.^26^ Targeting CaM and CaM‐dependent systems are considered useful strategies for potential cancer treatment interventions. It has achieved modest success by using chemical antagonists to inhibit CaM function or its targets, or by using interfering RNA to down‐regulate its expression alone or in combination with different chemotherapy drugs. CAMK1 is involved in multiple cell functions, including calmodulin binding, ATP binding, signal transduction, development and cell differentiation(GO database). Based on the GEPIA 2 database, Oncomine database and the HPA database, we demonstrated that compared to adjacent non‐cancer tissues, CAMK1 was highly expressed in PC tissues. The IHC data from the HPA database also revealed that the CAMK1 staining showed moderate to strong cytoplasmic immunoreactivity in most PC tissues. Furthermore, via analysis of prognostic value from the GEPIA 2 database and KM Plotter database, the expression of CAMK1 had significant prognostic correlation in the PC patients, high expression level of CAMK1 may correlate with a better prognosis in PC. Notably, the results from these bioinformatics databases were almost in agreement with our experimental results. In our TMA‐based IHC results, CAMK1 also mainly localized in the cytoplasm of PC cells and the CAMK1 staining showed moderate to strong cytoplasmic immunoreactivity in most of PC tissues. Moreover, the IHC score showed CAMK1 protein levels were up‐regulated in PC tissues compared to the corresponding non‐cancer tissues. The patients with CAMK1 higher expression also showed a superior OS compared to patients with CAMK1 lower expression.

The PPI information and pathway data enrichment analysis indicated that CAMK1 was mainly enriched in several KEGG pathways associated with aldosterone synthesis and secretion, oxytocin signalling pathway, et al, the candidate genes in these pathways included CALM1, CREB1, ATF1 and NOS3. Notably, these candidate genes were significantly up‐regulated in PC.

NOS3 locates on chromosome 7q36 and can encode endothelial nitric oxide synthase(eNOS), which produce nitric oxide (NO) in endothelial cells.^27^ NO is one of the smallest molecules in nature which plays key roles in cancer formation and progression.^28^, ^29^, ^30^ The generation of NO gradients around the blood vessels can normalize the tumour blood vessels and improve the response to anti‐cancer therapy.^31^ Recent research showed that the NanoNO, a nanoscale carrier that enables sustained NO release, can suppress tumour progression in combination with small‐molecule chemotherapy, macromolecular therapeutic agents.^32^ Evidence supporting that allosteric interaction of Ca2+/CaM complex with NOS is essential in NOS activation and NO release.^33^, ^34^ The Ca2+‐dependent pathway involving Ca2 + binding protein CaM can activate NOS3.^35^ Together these findings, we hypothesized that CAMK1 may also play important roles in regulation of NOS3 expression, although the precise mechanism underlying the association between CAMK1 and NOS3 requires further study. CREB is a nuclear transcription factor activated by phosphorylation at Ser133 by multiple serine/threonine (Ser/Thr) kinases. CREB can bind CREB‐binding protein (CBP) to initiate creb‐dependent gene transcription through phosphorylation. Present studies showed that CREB plays important roles in tumour initiation, progression and metastasis.^36^ Targeting CREB‐CBP interaction to inhibit CREB‐mediated gene transcription has become a hot spot in cancer treatment research.^37^ ATF1 plays a key role in tumour progression in a tumour‐specific manner. Overexpression of ATF1 has been found in various cancer. In lung cancer, ATF1 expression was associated with metastasis, tumour stage and poor prognosis, and^38^ in oesophageal cancer, ATF1 expression was correlated with poor differentiation, lymph node metastasis and early tumour invasion.^39^

In conclusion, it seemed that CAMK1 might be a promising biomarker for a better prognosis in PC patients, although the potential effect of CAMK1 expression on the biological function of PC and the reason for better prognosis remains to be further investigated. The PPI data only provided potential probabilities for interactions between genes based on different sources of information, and the underlying molecular mechanisms of CAMK1 in PC would be further explored.

## CONFLICT OF INTEREST

The authors declare that they have no known competing financial interests or personal relationships that would influence the work reported in this paper.

## AUTHOR CONTRIBUTIONS

**Yangyang Lei**: Formal analysis (lead); Conceptualization (equal); Data curation (equal); Writing‐original draft (equal). **Tianzhu Yu**: Methodology (supporting); Project administration (equal); Supervision (equal); Validation (equal). **Changyu Li**: Resources (equal); Writing‐original draft (supporting). **Jianke Li**: Validation (equal); Writing‐original draft (supporting). **Yicheng Liang**: Supervision (equal); Validation (equal). **Xinyuan Wang**: Writing‐original draft (supporting). **Yi Chen**: Project administration (equal); Supervision (equal). **Xiaolin Wang:** Funding acquisition; Project administration; Writing‐review & editing (equal).

## Supporting information

Fig S1

Fig S2

Table S1

## Data Availability

Some publicly available datasets were analysed in this study. The authors confirm that these data can be found here: http://gepia2.cancer‐pku.cn; www.oncomine.org; https://kmplot.com/analysis/; https://string‐db.org/cgi/input.pl; http://www.proteinatlas.org. These analyses of protein expression data of CAMK1 in the HPA database can be directly obtained from https://www.proteinatlas.org/ENSG00000134072‐CAMK1/pathology/pancreatic+cancer#ihc.
