# Supplementary figures and images for: Expression of CAMK1 and its association with clinicopathologic characteristics in pancreatic cancer
Source: J Cell Mol Med. 2020 Dec 20;25(2):1198–206. doi: 10.1111/jcmm.16188 (PMC7812292; doi:10.1111/jcmm.16188)

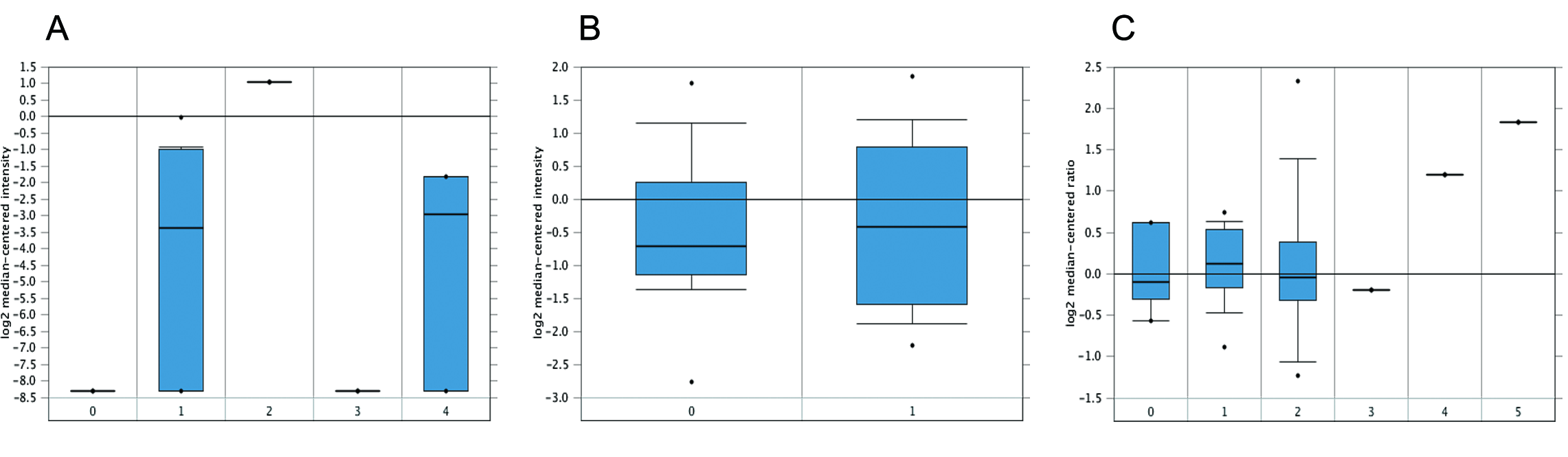

Supplement: Supplementary file 1 — Fig S1 [file JCMM-25-1198-s001.tif]

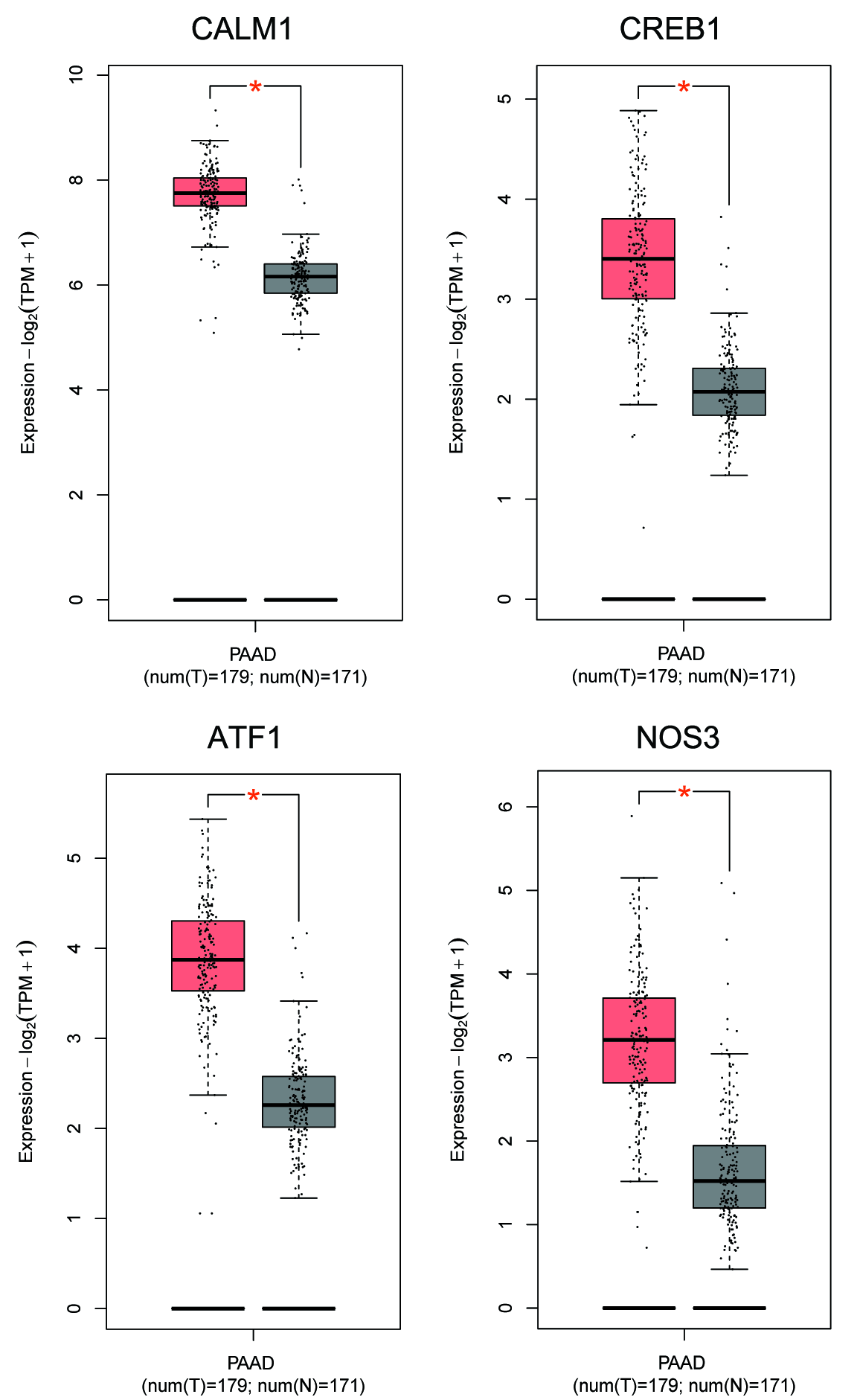

Supplement: Supplementary file 2 — Fig S2 [file JCMM-25-1198-s002.tif]
