# Supplementary material for: Expression of CAMK1 and its association with clinicopathologic characteristics in pancreatic cancer
Source: J Cell Mol Med. 2020 Dec 20;25(2):1198–206. doi: 10.1111/jcmm.16188 (PMC7812292; doi:10.1111/jcmm.16188)
Supplement: Supplementary file 3 — Table S1 [file JCMM-25-1198-s003.docx]

Supplementary Table 1

The KEGG pathway was constructed to explore the potential biological value of CAMK1 in pancreatic cancer. False discovery rate(FDR)＜.01.

| Pathway | Description | Gene | False discovery rate |
| --- | --- | --- | --- |
| [hsa04925](https://www.kegg.jp/kegg-bin/show_pathway?hsa04925) | Aldosterone synthesis and secretion | CALM1, CAMK1, CREB1, ATF1 | 1.31e-05 |
| [hsa04921](https://www.kegg.jp/kegg-bin/show_pathway?hsa04921) | Oxytocin signaling pathway | CALM1, NOS3, CAMK1 | 0.0019 |
| [hsa04915](https://www.kegg.jp/kegg-bin/show_pathway?hsa04915) | Estrogen signaling pathway | CALM1, NOS3, CREB1 | 0.0019 |
| [hsa04022](https://www.kegg.jp/kegg-bin/show_pathway?hsa04022) | cGMP-PKG signaling pathway | CAML1, NOS3, CREB1 | 0.0019 |
| [hsa05031](https://www.kegg.jp/kegg-bin/show_pathway?hsa05031) | Amphetamine addiction | CALM1, CREB1 | 0.0084 |
